# The essential role of aggregation for the emulsifying ability of a fungal CYS-rich protein

**DOI:** 10.1007/s00253-024-13182-7

**Published:** 2024-06-03

**Authors:** Rossana Pitocchi, Paola Cicatiello, Anna Illiano, Carolina Fontanarosa, Federica Spina, Giovanna Cristina Varese, Angela Amoresano, Alessandra Piscitelli, Paola Giardina

**Affiliations:** 1 Department of Chemical Sciences, University of Naples Federico II, Via Cintia, Naples, 80126 Italy; 2 Department of Life Sciences and Systems Biology, University of Turin, Viale P.A. Mattioli 25, Turin, 10125 Italy

**Keywords:** Fungal proteins, Bioemulsifier, Aggregation, Plastic-degrading fungi, CFEM domain

## Abstract

**Abstract:**

Biosurfactants are in demand by the global market as natural commodities suitable for incorporation into commercial products or utilization in environmental applications. Fungi are promising producers of these molecules and have garnered interest also for their metabolic capabilities in efficiently utilizing recalcitrant and complex substrates, like hydrocarbons, plastic, etc. Within this framework, biosurfactants produced by two *Fusarium solani* fungal strains, isolated from plastic waste-contaminated landfill soils, were analyzed. Mycelia of these fungi were grown in the presence of 5% olive oil to drive biosurfactant production. The characterization of the emulsifying and surfactant capacity of these extracts highlighted that two different components are involved. A protein was purified and identified as a CFEM (common in fungal extracellular membrane) containing domain, revealing a good propensity to stabilize emulsions only in its aggregate form. On the other hand, an unidentified cationic smaller molecule exhibits the ability to reduce surface tension. Based on the 3D structural model of the protein, a plausible mechanism for the formation of very stable aggregates, endowed with the emulsifying ability, is proposed.

**Key points:**

*• Two Fusarium solani strains are analyzed for their surfactant production.*

*• A cationic surfactant is produced, exhibiting the ability to remarkably reduce surface tension.*

*• An identified protein reveals a good propensity to stabilize emulsions only in its aggregate form.*

**Supplementary Information:**

The online version contains supplementary material available at 10.1007/s00253-024-13182-7.

## Introduction

Surfactants, thanks to their amphiphilic nature, display different properties such as detergency, solubilization, and lubrification, stabilizing and foaming capacity, and additionally, they can form phase dispersion (Kaczorek et al. 2018). For these reasons, they are useful in a great variety of application fields like medical, food and beverages, cosmetics, agriculture, detergent, textiles, and petrochemical production. Very often they are petroleum-derived molecules, and hence non-renewable, difficult to dispose and can be toxic if accumulated in the soil/sea (Ng et al. 2022). Biosurfactants (BS) on the other hand, are amphiphilic molecules produced by microorganisms, like bacteria, yeasts, and fungi. They possess the same characteristics as synthetic surfactants, with the added value of being eco-friendly, biodegradable, and less toxic. These amphiphilic compounds can be classified based on different characteristics, like charge, chemical structures, or molecular weight (Nikolova and Gutierrez 2021). Low molecular weight microbial products are generally referred to as BS as they can lower interfacial tension, some examples are lipopeptides, sugars, amino acids, and fatty acids. High molecular weight biosurfactants, such as proteins, lipoproteins, and hetero/lipopolysaccharides are best classified as bioemulsifiers (BE) since they can efficiently emulsify immiscible liquids, even at low concentrations (Fenibo et al. 2019). Nonetheless, it can be assumed that one kind of activity does not preclude the other (Pitocchi et al. 2020). Microorganisms may release biosurfactants as an adaptive response to the increasing presence of hydrocarbon-based compounds in the environment to use them as a carbon source (Perfumo et al. 2010). Among BS-producing microorganisms, bacteria are the most characterized (Shakeri et al. 2020; Pardhi et al. 2022). The reason why filamentous fungi are less exploited is probably due to their slower growth and the lower yields generally achievable. However, it is important to find other promising biosurfactant-producing microorganisms in order to increase the variability of these biomolecules for large-scale production and, additionally, to reduce the use of some of these microbes, that are known human pathogens (e.g., *Pseudomonas aeruginosa*, *Candida*, and *Bacillus*) (Nikolova and Gutierrez 2021). Filamentous fungi examined so far were great BS sources (Bhardwaj et al. 2013). They have developed an extraordinary ability to adapt to changing environments and tolerate several types of pollutants. In fact, fungi can extend through substrates in their search for nutrients with their filamentous network structure, exploring and growing in places that are more difficult to reach for other microorganisms (Sánchez 2020). In this sense, fungi have promising practical applications to remediate environmental pollution, even plastic-based pollution. Fungi owe their huge potential to characteristics like their enzymatic systems, their ability to produce surface-active proteins (like hydrophobins, HPBs) for the attachment of hyphae to hydrophobic substrates, and their cellular ability to penetrate three-dimensional substrates (Sanchez 2020). Plastic biodegradation by fungi involves several steps, however, attachment on the polymer surface is the initial step and is mainly mediated by HPBs (Hektor and Scholtmeijer 2005). It has been reported that surface active proteins, particularly HPBs or HPBs-like proteins, are effective BS (Askolin et al. 2006; Cicatiello et al. 2019; Pitocchi et al. 2023) giving the same or even better performances than synthetic surfactants. Two strains of *Fusarium solani* isolated in landfill soils by the Mycoteca Universitatis Taurinensis (MUT) were selected for their ability to reduce surface tension. Spina et al. (Spina et al. 2021) reported that *F. solani* possesses a great ability to grow on polystyrene (PE). In this paper, we analyze the BS produced by these strains and demonstrate that, surprisingly, aggregates formed by an unknown protein are responsible for the high capacity of emulsion stabilization.

## Materials and methods

Fungi were supplied by the Mycoteca Universitatis Taurinensis (MUT). *F. solani* (MUT 4426) and *F. solani* (MUT 6181) were isolated in landfill soils (Emilia Romagna, IT) contaminated with different substances such as plastic materials and selected for their ability to reduce surface tension (Spina et al. 2021).

### Fungal growth

Fungal strains were maintained through periodic transfer on agar plates at 25° C, using malt extract agar medium. Mycelium discs (10 mm diameter) were taken from the margin of the actively growing colonies and inoculated in 250 ml flasks containing the medium (0.3 g/L KH_2_PO_4_, 0.3 g/L MgSO_4_, 3 g/L NaNO_3_, 2 g/L yeast extract) enriched with two different amount of olive oil, precisely 5% and 1% (v/v). The flasks were inoculated in triplicates and incubated in the dark at 25 ° C for 10 days.

### BS extraction

At the end of incubation time, mycelia were separated from the cultural broths with a Whatman paper 3 mm filters. The culture broth was centrifuged (4250 xg for 15 min) and filtrated using a Stericup GP vacuum filtration system (Merck KGaA, Darmstadt, Germany). BS were extracted from the culture broth by adding MetOH: chloroform at a 1:1:0.5 v/v ratio, respectively. The sample was then centrifuged (1420 xg for 1 h) and BS were recovered in the MetOH -water phase. The extract was then concentrated to the desired volume using a Rotavapor (Hei-Vap Core, Heidolph KG, Schwabach, Germany).

Protein concentration was evaluated using the BioRad Protein Assay kit (Hercules, California, USA) using bovine serum albumin as standard.

### SDS-PAGE

Samples were first treated with Sample Buffer 4 × (2% sodium dodecyl sulfate (SDS), 80 mM Tris-HCl pH 6.8, 10% glycerol, 0.002% bromphenol blue, 0.05 M dithiothreitol (DTT), and then they were further denatured boiling them at 100°C for 10 min. Electrophoretic runs were performed loading the samples on 12.5% acrylamide gels (12.5% acrylamide, 0.1% w/v bis-acrylamide, 0.4 M Tris-HCl pH 9.2, 0.1% w/v SDS, 0.1% w/v ammonium persulfate (APS), 0.001% v/v tetramethyl ethylenediamine (TEMED)) and soaking it in 0.1 M Tris-glycine pH 8.3. Silver stain coloration involves multiple steps and solutions reagent: first, in pH 6.5 solution A (0.5% glutaraldehyde, 0.1 g/L sodium tiosulphate, 30% ethanol, 0.4 M NaCl) was incubated for 1 hour followed by 3 water washings of 10 min each. Then the gel in a solution B (AgNO_3_ 1 g/L and formaldehyde 125 µl) was incubated for 30 min. During the last step the gel was stained by the solution C (3% Na_2_CO_3_ and formaldehyde 0.015%). When not specified, gels were dyed with Colloidal Coomassie G-250, BioRad. In the case of harsher denaturation treatment, samples were diluted in 4 M urea, 0.1 M DTT and then incubated at 60°C for 1 h. At the end of the incubation time, 0.5 M iodoacetamine (IAM) was added to the solution and incubated in the dark for 45’. To stop the reaction, 10% formic acid was added. Protein was then recovered using methanol-chloroform-water extraction (3:1:1 v/v).

### Thin-layer chromatography (TLC)

Silica gel on TLC aluminum foil was used as a stationary phase. A mixture of toluene-chloroform-acetone (7:2:1) was used as the mobile phase, phosphomolybdic acid (10% w/v in pure ethanol) and sulfuric acid (3% v/v in 10% ethanol) were used as a color developer for the detection of lipids and sugars respectively, through dipping and heating. 20 µl of the extracted broths (MetOH 50%) were loaded on TLC with multiple deposition of 2 µl; 10% olive oil in MetOH 50% was used as a control.

### Surface activity

Drop collapse consists of depositing a sample drop onto a hydrophobic surface (parafilm M®, Amcor, Melbourne, Australia), and then evaluating the collapsing area. BS solutions should spread on the surface more extensively than water; pictures were acquired after 30 min.

The surface tension, γ, of the extracts was measured with a Sigma 70 tensiometer (KSV, Stockholm, Sweden) using the Du Noüy ring method (Russo et al. 2017). γ was related to the force required to lift the ring from the surface of the air/liquid interface. 7 ml of sample were added to the vessel and allowed to balance 5 min prior to measuring surface tension. Water has been used to calibrate the tensiometer (72 mN/m).

### Emulsification test

The emulsification capability of each sample was investigated. 2 ml of Dectol (a mix of decane-toluene 65:35, v/v) were added as emulsifying agent to 1 ml of each surfactant protein suspended in 10 mM phosphate buffer (pH 7.0) in 5 ml glass vials (Blesic et al. 2018). This mixture was homogenized using the IKA T-10 Basic Ultra Turrax Homogenizer (IKA-Werke GmbH, Staufen, Germany) for 3 min, then the stability of the emulsions during the time was evaluated. Stability is reported as emulsification index after 24 h, E_24_.

$${E}_{24}=\frac{foam\;layer\;height}{total\;volume\;height} x 100$$

Proteins samples, 0.05 mg/mL, were incubated with Proteinase K (from *Tritirachium album*, Sigma-Aldrich, St. Louis, MO, USA) 1 mg/mL at 37 °C for 30 min. Then, an emulsification test was performed as described above.

### Protein identification by Mass Spectrometry (MS)

Protein identification by mass spectrometry was performed on Coomassie Blue stained bands excised from mono-dimensional gels. Protein bands corresponding to proteins of interest (molecular mass about 10 kDa), were excised from gel, destained by washes with 0.1 M NH_4_HCO_3_ pH 7.5 and acetonitrile, reduced for 45 min in 100 µL of 10 mM dithiothreitol, 0.1 M NH_4_HCO_3_, pH 7.5, and carboxyamidomethylated for 30 min in the dark by the addition of 100 µL of 55 mM iodoacetamide (IAM) dissolved in the same buffer. Enzymatic digestion was performed and was then analyzed with 6520 Accurate-Mass Q-TOF LC/MS system (Agilent Technologies, Palo Alto, CA, USA) equipped with a 1200 HPLC (high-performance liquid chromatography) system and a chip cube (Agilent Technologies). The acquired MS/MS spectra were transformed in Mascot Generic format (mgf) and used to query the NCBInr database, with taxonomy restriction to Fungi (http://www.ncbi.nlm.nih.gov).

### UV-Vis analysis

Fluorescence spectra were recorded at 25 °C with a HORIBA Scientific Fluoromax-4 spectrofluorometer (Horiba, Rome, Italy). Slits were set to 3 and 6 nm spectral band-passes in excitation and emission monochromators, respectively. Tryptophan fluorescence emission was observed using λ_exc_ of 280 nm (emission range 300–500 nm); fluorescence emission of the putative fluorophore compounds was observed using λ_exc_ of 325 nm (emission range 350–550 nm).

### Protein purification

5 mg of total proteins were loaded on a Superdex 75pg HiLoad column (Merck KGaA, Darmstadt, Germany), with a flow of 0.3 ml/min and monitoring the wavelength at 280 nm. 10 mM sodium phosphate with 150 mM NaCl was used as elution buffer, fractions of 1 ml were collected and then analysed. Successive chromatography purification was performed using anionic exchange column. 0.3 mg of sample were first dialyzed with 10 mM sodium phosphate buffer 10 mM (RC dialysis tube, 3.5 kDa cut-off, Merck KGaA, Darmstadt, Germany). Emulsification ability of the gel filtration peaks was verified after this process, and then the sample was loaded on HiTrap Q XL, 1 ml column, and eluted using a linear gradient with 0.5 M NaCl as final salt concentration, 0.5 M using a linear gradient at 1 ml/min flux and monitoring the absorbance at 280 nm. Fractions were collected and further analysed.

### Liquid chromatography with tandem mass spectrometry (LC-MS/MS) analysis

Samples were diluted 1:10 in MetOH, filtered and centrifuged (10,000 rpm for 10 min). The supernatant was then directly transferred into HPLC auto sampler and 1 µl of supernatant was analysed by using an AB-sciex 5500 QTRAP® system with a HPLC chromatography system Exion LC™ (Agilent Technologies, Palo Alto, CA, USA). The mobile phase was generated by mixing eluent A (0.1% formic acid in water) and eluent B (0.1% formic acid in acetonitrile) and the flow rate was 0.2 mL/min. Chromatographic gradient was from 20 to 90% in 4 min, hold for 2 min, then return to 20% in 1 min. Tandem mass spectrometry was performed using Turbo VTM ion source operated in positive ion mode, and the multiple reaction monitoring (MRM) mode was used for the selected analytes. Qualitative analysis and identification of the phenolic compounds contained in samples were carried out by using LC-MS/MS in negative (ESI-) ionization modes. A set of targeted molecules were explored by mass spectrometry in multiple reaction monitoring as reported in the supplementary section (Supplementary Table S1) taking advantage of high performances of triple quadrupole mass spec. For quantification of analytes, standard calibration curves for the selected set of molecules, were constructed by plotting peak areas against concentration (µg/L), and linear functions were applied to the calibration curves. The coefficients of determination (R2) were greater than 0.99 for all analytes. The extracted mass chromatogram peaks of metabolites were integrated using Skyline software for data processing. For each molecule specific transitions precursor ion/fragment ion were selected (supplementary Table S1). The LCK331/LCK332/LCK334 (Hach Lange GMBH, Linate, Italy) tests were used for the determination of anionic, cationic, and non -ionic surfactants, through a titration with bromine blue phenol. The concentration is determined by spectrophotometric analysis.

## Results

### Fungal growth

**Fig. 1:**
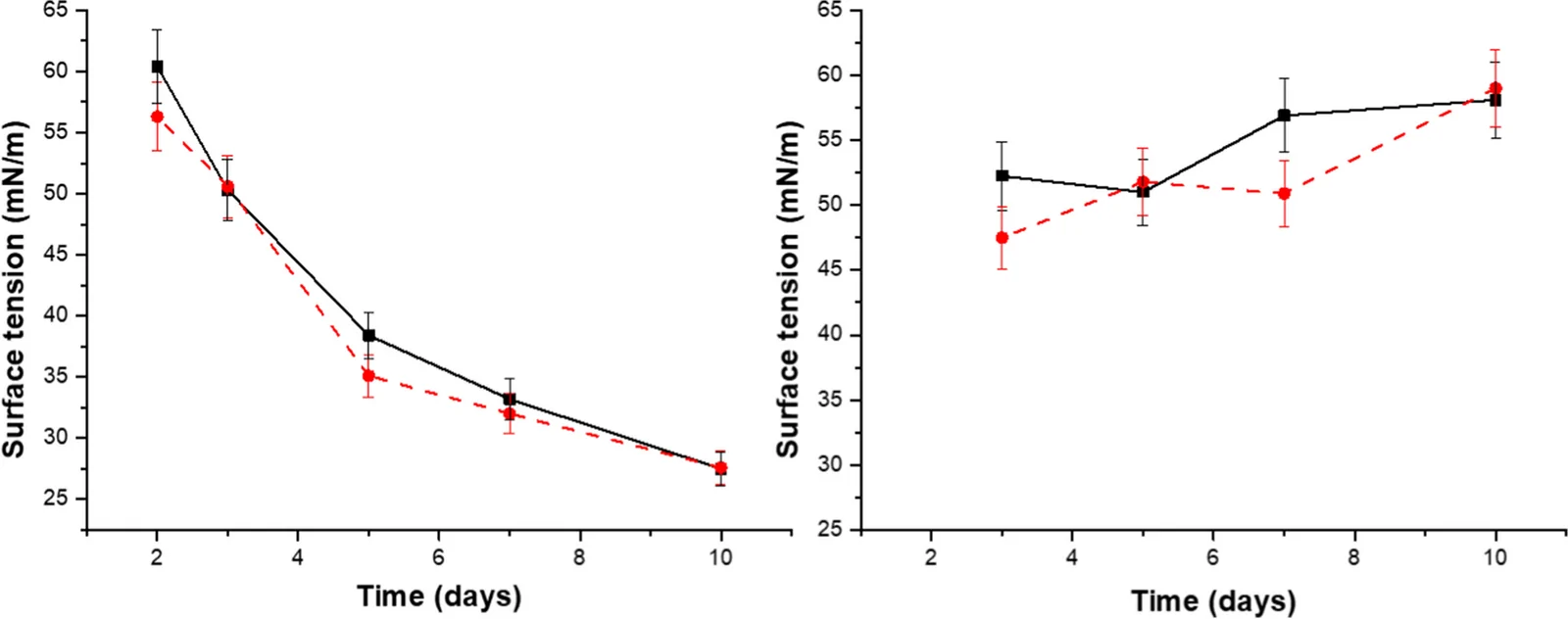
Surface tension decrease as a function of days of growth. On the left, fungal growth performed in the presence of 1% of olive oil; on the right, in the presence of 5% olive oil. Solid black line, for MUT4426; red dashed line, for MUT6181

*F. solani* (MUT 4426 and MUT 6181) were grown in the presence of olive oil as a carbon source, to stimulate BS production. Two different amounts of olive oil were used, 1% and 5% (v/v), to better exploit BS induction and surface tension was monitored during the fungal growth in the two conditions. Culture media, enriched with the same amounts of olive oil, used as a control, led to a minimal surface tension decrease compared to that of water (68 mN/m versus 73 mN/m). Figure 1 shows that the lowest value is achieved when 5% olive oil is present in the culture media. On the other hand, no relevant amount of BS seemed to be produced when 1% of olive oil was added, as verified by the constant and high value of surface tension in this condition.

Mycelia were separated from the culture broth, freeze-dried, washed with hexane to remove adhered oil, and weighed. A much higher amount of fungal biomass (about fourfold for both strains) was obtained when culture broth was implemented with 5% olive oil. Thus, 5% olive oil and 10 days of growth were hence used as the best-growing conditions for all the successive experiments. BS production was also checked by emulsion tests in the presence of Dectol, finding neglectable results in both tested conditions.

### BS characterization and protein identification

**Fig. 2:**
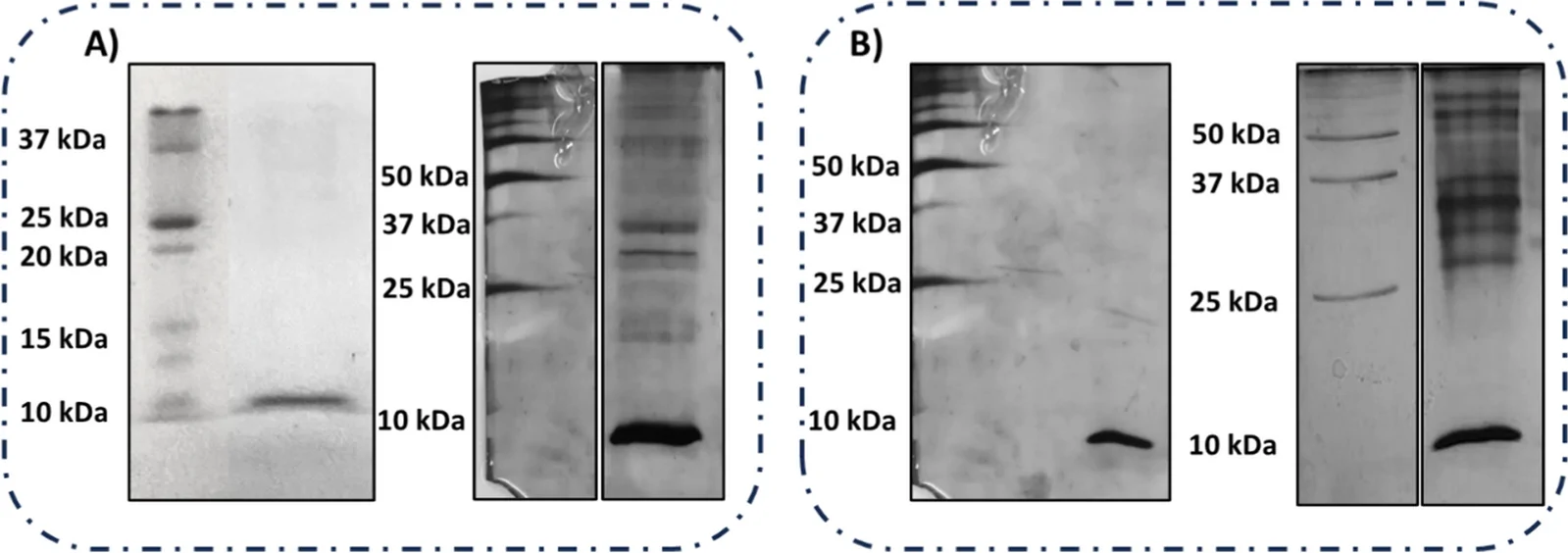
Silver stained SDS-PAGE of raw extracts of MUT4426 (A) and MUT6181 (B) before (left) and after (right) concentration

To purify BS produced by the strains, a commonly used MetOH -chloroform extraction protocol was adopted. To determine the chemical nature of the compounds present in these raw extracts, thin-layer chromatography was used, as a fast and simple technique (Supplementary Fig. S1). No lipids or sugars were detectable by the staining methods used. On the other hand, protein concentrations of both extracts were evaluated and found to be 60¸80 µg/ml, hence the SDS-PAGE was carried out (Fig. 2). Only one protein band was visible in each of the raw extracts loaded on the SDS PAGE. These bands were excised and submitted to a classical proteomic approach to identify proteins. The same protein could be confidently identified in the respective protein bands as C7Z2B1 from *F. solani*, a “common in fungal extracellular membrane (CFEM)” domain-containing protein. The identified peptides and the protein sequence are reported in Fig. S2A, B. Analysis of the protein aminoacidic sequence showed an isoelectric point of 7.45 and a significant hydrophobic character (grand average of hydropathy, GRAVY + 0.487) (Gasteiger et al. 2005) where three sequence portions showed a score > 1 (Kyle Doolittle scale, Fig. S2C). Eight Cys-residues are usually found in the CFEM domain, while the protein C7Z2B1 displays an even higher Cys content (10 Cys).

The raw extracts were distilled to remove MetOH and concentrated (tenfold the initial culture broth volume). Afterwards, the same amounts of proteins (1 µg) were loaded again on the gel, and surprisingly other protein bands at higher molecular weight could be detected (Fig. 2).

The appearance of several new bands at high molecular weights may suggest that formation of aggregates occurs after sample concentration. This phenomenon is not related to denaturation of these proteins since their structures, determined by circular dichroism analysis, did not change when the proteins were purified by ultrafiltration and dialysis, a protocol that led to a scarce protein yield (data not shown).

### Emulsifying and surface activity of the raw extracts

At first surface activity of the concentrated raw extracts was qualitatively tested using drop collapse test. This first measurement validated the hypothesis that water-soluble surface-active compounds were present in the extracts, as the drop shape of the samples on a hydrophobic surface was flattened compared to the control. Quantitative measurements, using the tensiometer, confirmed the lower surface activity of these extracts, showing surface tension values of about 45 mN/m for both extracts. Emulsification tests were also carried out, revealing that both samples can efficiently stabilize emulsions exhibiting an E_24_ value of about 80%. The different behavior of these raw extracts respect to that of the culture broths (no emulsifying ability) can be ascribed to the higher concentration.

**Fig. 3:**
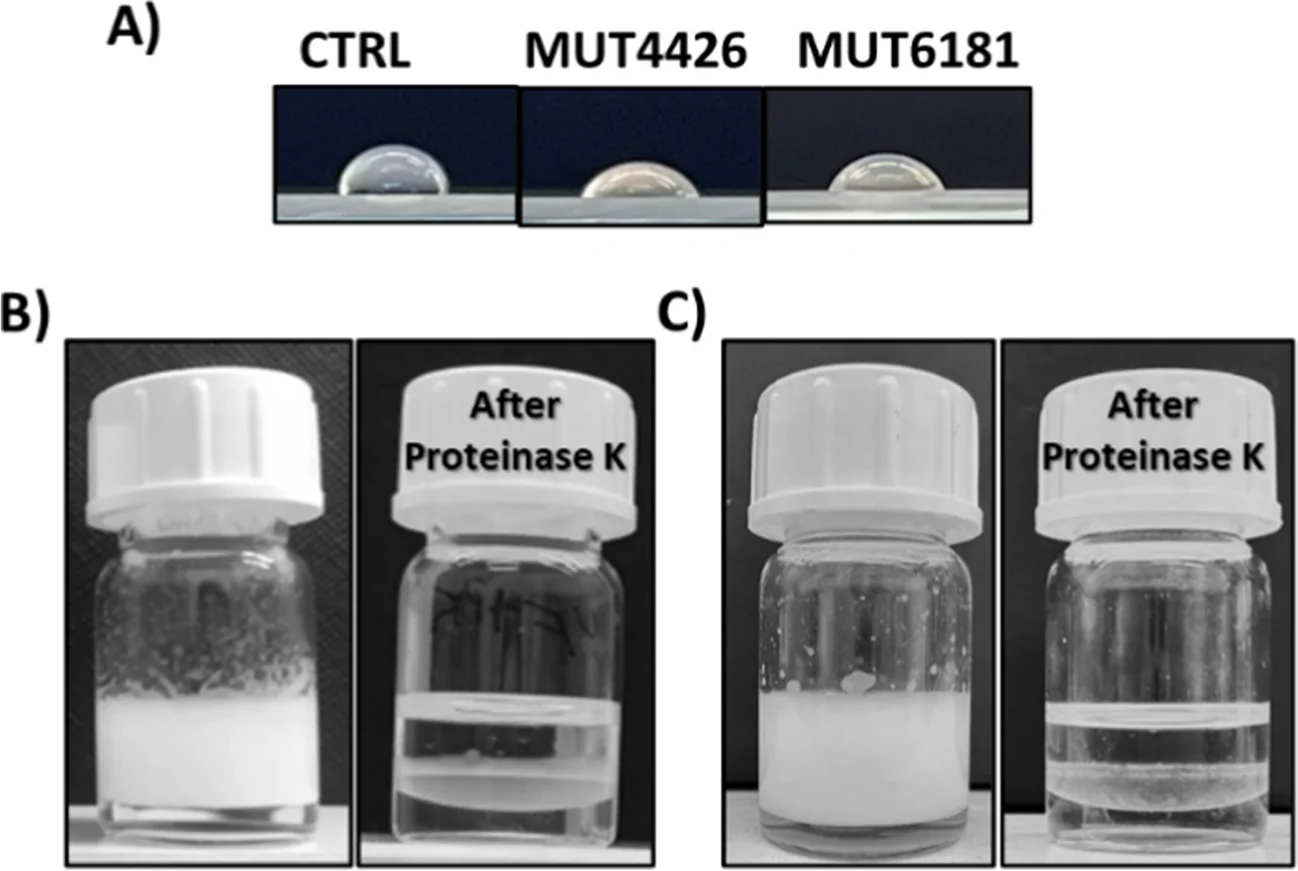
**A** Drop collapse test on a hydrophobic surface (Parafilm) of both concentrated raw extracts. Pictures were taken after 30 min from drop deposition, water is shown as a control (CTRL). **B**, **C **Emulsification tests of concentrated raw extracts of MUT4426 (**B**) and MUT6181 (**C**), before (left) and after (right) the hydrolytic treatment with Proteinase K. Images were taken 24 h after emulsification

**Fig. 4:**
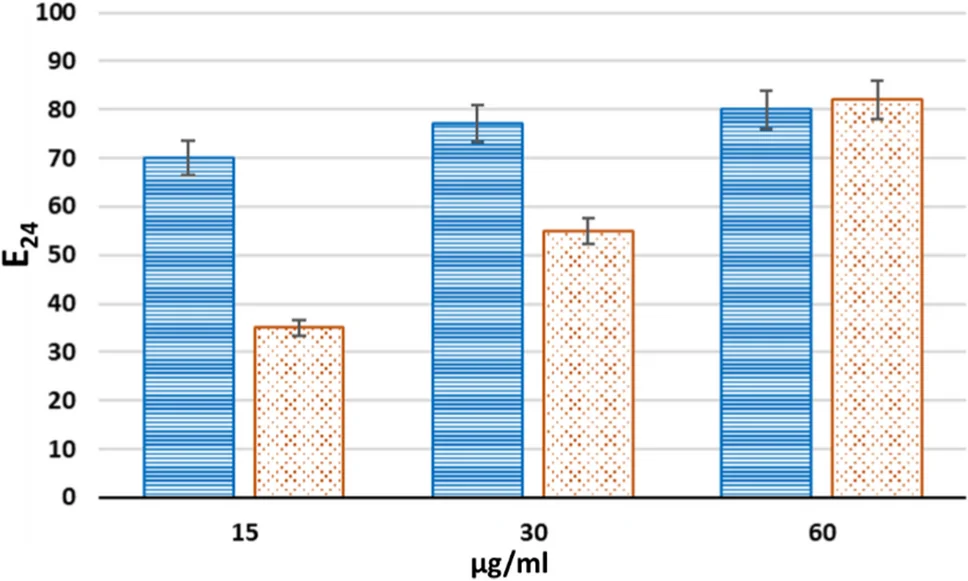
E_24_ values of the two concentrated raw extracts as a function of protein concentration. Blue dashed columns represent the MUT6181, and orange dotted columns MUT4426. Error bars are calculated as standard deviation on at least three technical replicates

A hydrolytic treatment with Proteinase K was performed to ensure that the observed low surface tension and emulsifying capability were related to the presence of proteins. Indeed, after this enzymatic treatment, no emulsion was observed, thus proving the crucial role of proteins in forming and stabilizing emulsions (Fig. 3). On the other hand, reduction of the surface tension was still achieved even after the hydrolytic treatment. These results indicate that the protein components in the extracts are responsible for the emulsifying ability, while the surface tension reduction should be due to different compounds. Hence, further drop collapse tests and emulsification tests as a function of protein concentrations were performed (Fig. 4). Experiments revealed that both samples can efficiently stabilize emulsions reaching a maximum value of E_24_ of 80% at the highest concentration tested. Emulsions at higher protein concentration were not tested because of the rapid protein precipitation occurring when further concentrated. The samples from MUT6181 showed a higher emulsification index at low concentration, while the E_24_ of the samples from MUT4426 increased slower as a function of concentration.

### Protein purification

**Fig. 5:**
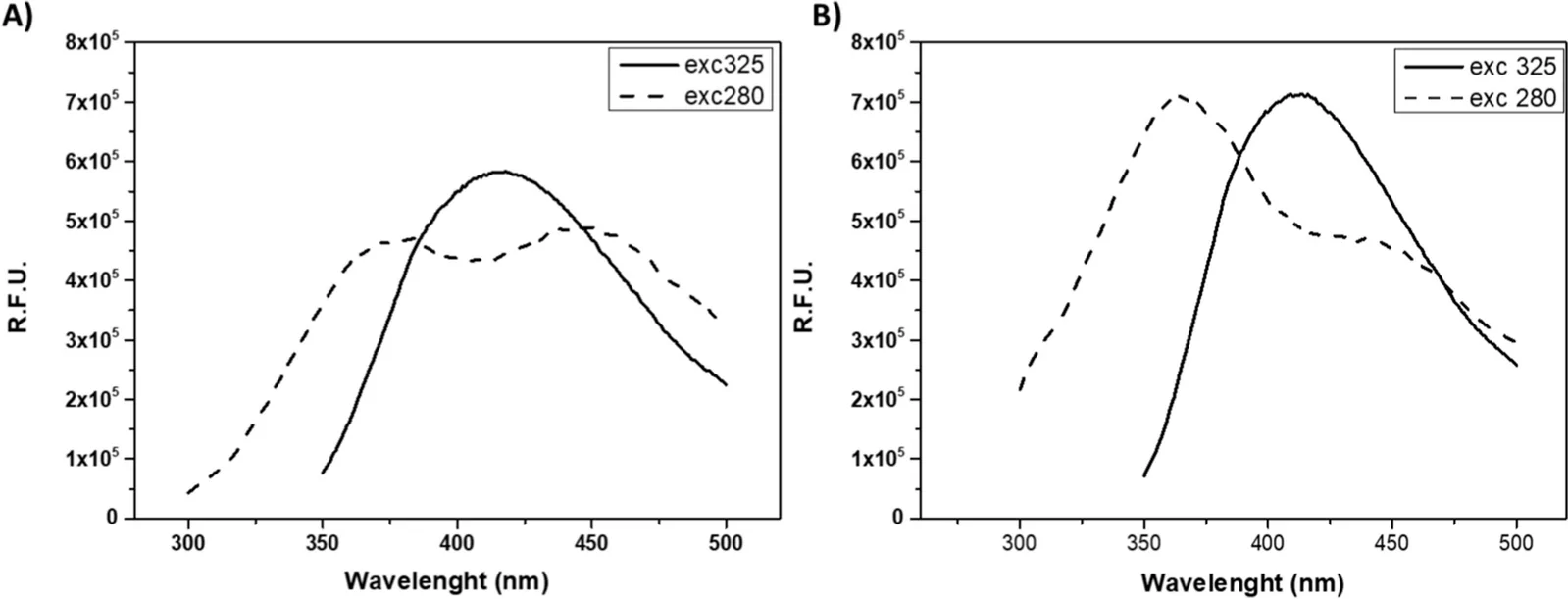
Fluorescence emission spectra of concentrated raw extracts of MUT4426 (**A**) and MUT6181 (**B**) at λ_exc_ 280 nm (dashed line) and λ_exc_ 325 nm (solid line). R.F.U. is the reference fluorescence unit

Spectrophotometric analyses were carried out on the two samples. Absorption spectra of both samples surprisingly showed another intense peak at 325 nm, besides the peak corresponding to Trp (280 nm). Thus, fluorescence spectra were recorded using 280 nm and 325 nm as λ_exc_ (Fig. 5).

When the λ_exc_ was 280 nm, two peaks were detected: a first one around 360 nm, corresponding to the Trp emission, the second one, near 450 nm suggesting the presence of a fluorophore different from Trp. When λ_exc_ was 325 nm, indeed, an intense emission peak appeared at λ around 430 nm. The spectra at 280 nm λ_exc_ of the two samples, exhibited similar patterns, except for the relative intensity of the two peaks, indicating different abundance of the protein respect to the other unknown fluorophore.

**Fig. 6:**
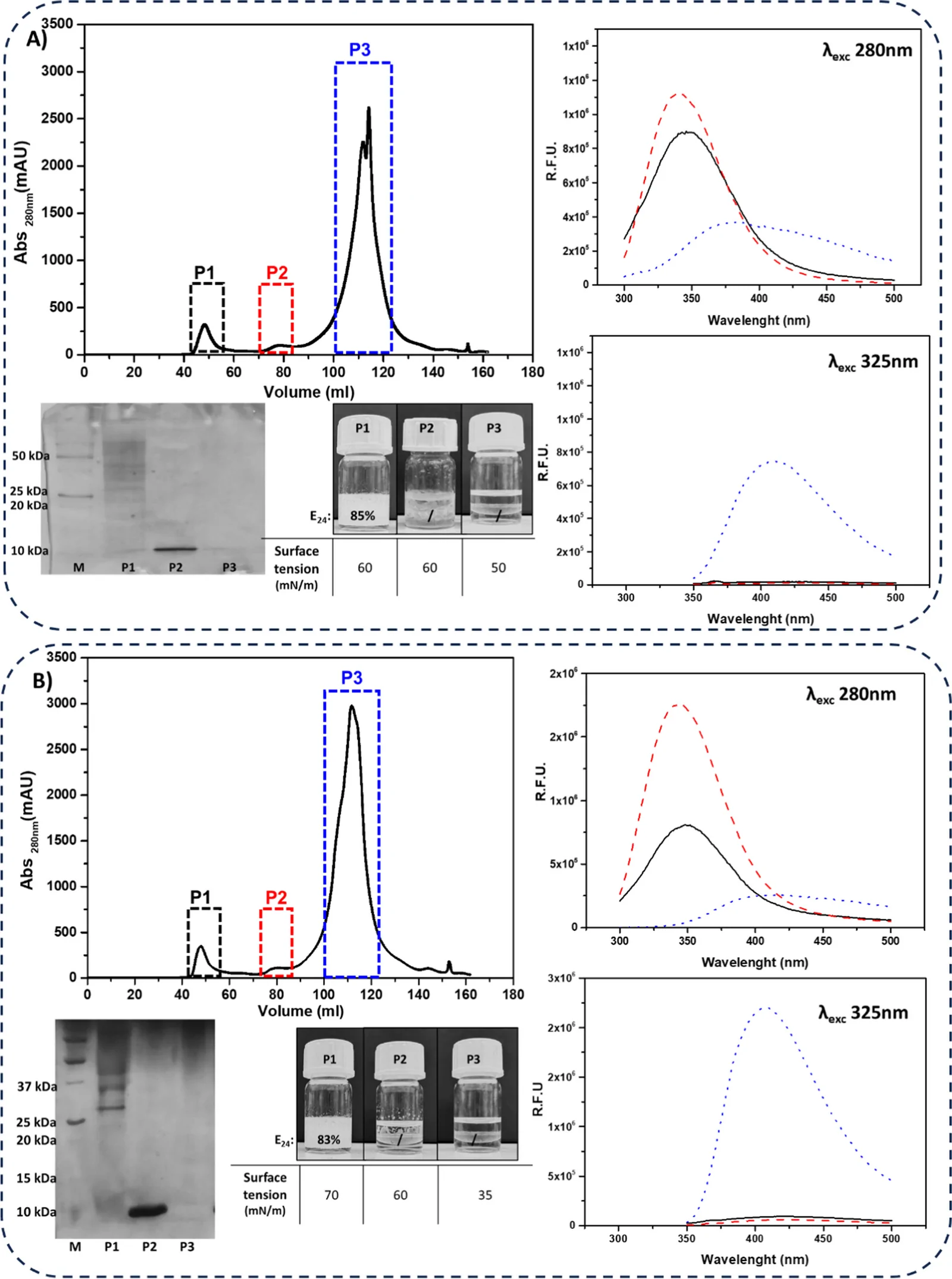
Purification and characterization of the raw extracts of MUT4426 (**A**) and MUT6181 (**B**). Top left: Chromatographs of gel filtration columns, with collected peaks highlighted in black, red, and blue named P1, P2, and P3 respectively. Fluorescence emission spectra of each peak, at 280 nm λ_exc_ (top right), and 325 nm λ_exc_ (bottom right); P1 black solid line, P2 red dashed line, P3 blue dotted line. R.F.U. is the reference fluorescence unit. SDS-PAGE, emulsification tests, surface tension values of each peak (bottom left)

Intending to understand if the fluorophores are free or protein-bound, and the role of each component, a gel filtration chromatography was tried out. Each raw extract was concentrated fivefold just before loading to avoid protein precipitation. The chromatograms showed three main peaks for both samples, that were collected and characterized as shown in Fig. 6.

As reported in Fig. 6, very similar results were obtained from the two chromatography experiments. In peak 1 (P1) of both samples, protein concentration was two-fold compared to that one of peak 2 (P2), about 0.1 mg/ml and 0.05 mg/ml respectively, whereas no protein at all was detected in P3. SDS-PAGE analysis, moreover, highlighted the presence of the single protein band near 10 kDa in P2, such as that one previously identified in the raw extract. In P3 no protein band was detected, while multiple protein bands were visible in P1 whose molecular weights were higher than 25 kDa. Analysis of fluorescence emission of these fractions showed that Trp emission was only observed for P1 and P2 and not for P3 (black spectra in Fig. 6A-B), as expected. On the other hand, the emission peak near 450 nm relative to the other putative fluorophore, (λ_exc_ 325 nm, red spectra in Fig. 6A-B), was only present in P3.

Moving to the analyses of surface and emulsification activity of the chromatographic fractions, results indicated that only P1 retains the ability to stabilize emulsions, while unexpectedly P2 does not (at the same protein concentrations, 0.05 mg/ml). On the other hand, the decrease in surface tension was only related to P3. These observations seem to indicate that emulsification and surface activity are linked to two different components; the former can be attributed to high molecular weight proteins and the latter to unknown smaller molecules, also responsible for the fluorescence emission at 450 nm.

Considering that chromatograms and relative peak characterizations revealed that the two strains exhibit very similar behavior, herein after results are only reported for MUT6181.

To further study the nature of the compounds eluted as P3, several analyses were performed. Using kits for the analysis of anionic, cationic and non-ionic surfactants only a cationic surfactant was found in this sample (9 mg/L), confirming what was previously determined by measuring the surface tension.

The analysis carried out with LC-MS/MS in MRM mode, showed the presence of different polyphenols (Supplementary Table S2). It is worth noting that the most represented compound among those explored was naringenin in P3 (about 5 mg/L). Anyway, examining the literature data on this compound, naringenin should be not responsible neither for the observed fluorescence emission of P3 (Uivarosi et al. 2016; Li et al. 2021) nor for the surface tension decrease.

As P1 concerns, the unique fraction showing emulsification activity, SDS-PAGE analysis revealed the presence of multiple bands. After the hydrolytic treatment with Proteinase K, no emulsion was formed (Supplementary Fig. S3). Hence, P1 sample underwent an anionic exchange chromatographic step, to try to purify the protein responsible for the emulsification activity.

**Fig. 7:**
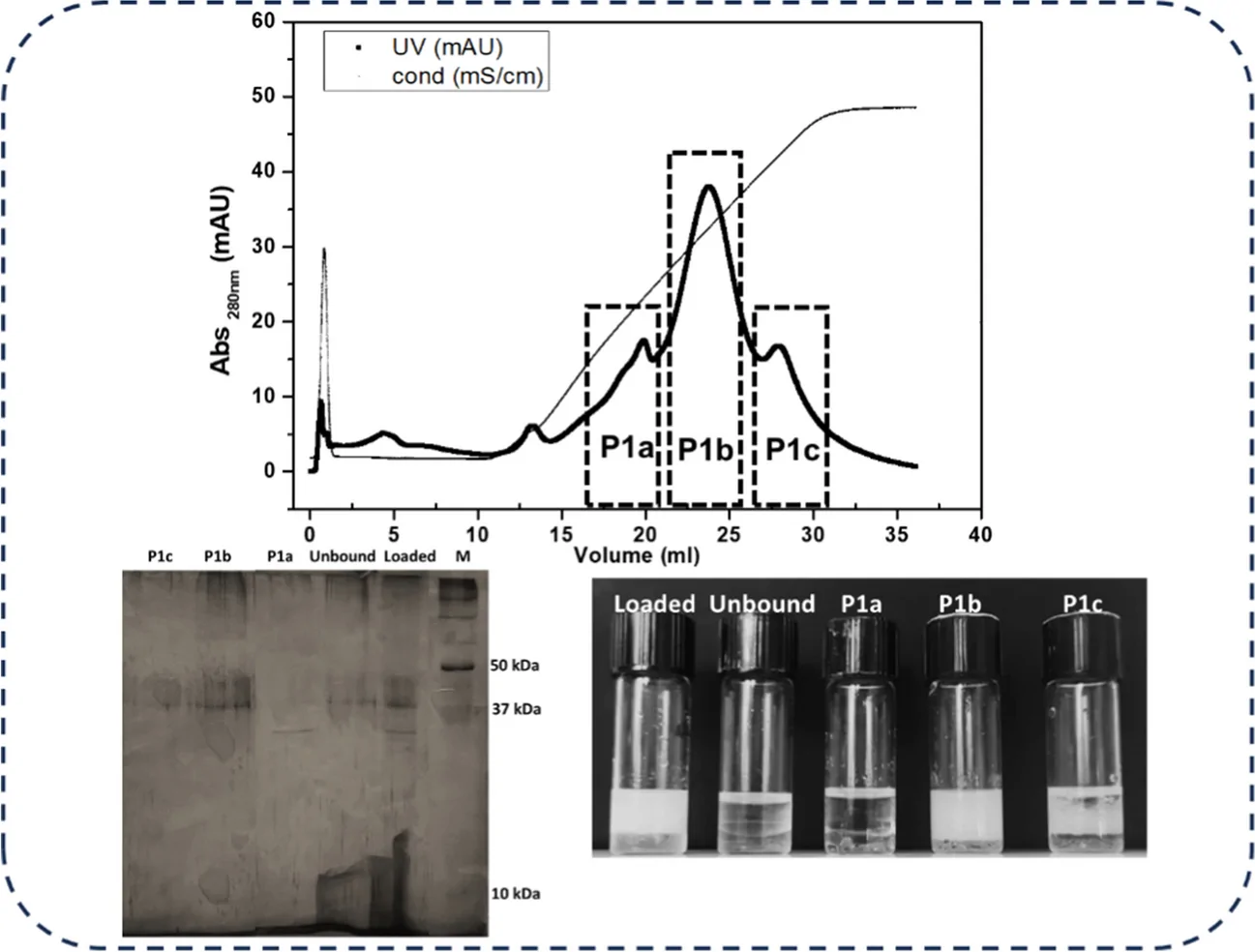
Chromatogram obtained from anionic exchange separation of P1 sample; peaks collected are highlighted in dotted boxes. SDS-PAGE analysis (bottom left) and emulsification tests of the loaded sample, unbound fraction, and the collected peaks (bottom right) are shown

Three different fractions were collected and named P1a, P1b, and P1c. Only P1b retained an emulsification ability similar to that of the loaded sample, the SDS-PAGE shows again multiple bands relating to this peak (Fig. 7). Hence this sample was subjected to proteomic analysis (Supplementary Fig. S4). The presence of the C7Z2B1 protein in this sample led to the hypothesis that aggregation phenomena could occur, as a consequence of the concentration step performed before the gel filtration, moreover the aggregates should not be effectively broken during the SDS-PAGE sample preparation. According to this hypothesis, emulsification activity should be only ascribable to aggregates of that protein, and not to its monomeric form.

**Fig. 8:**
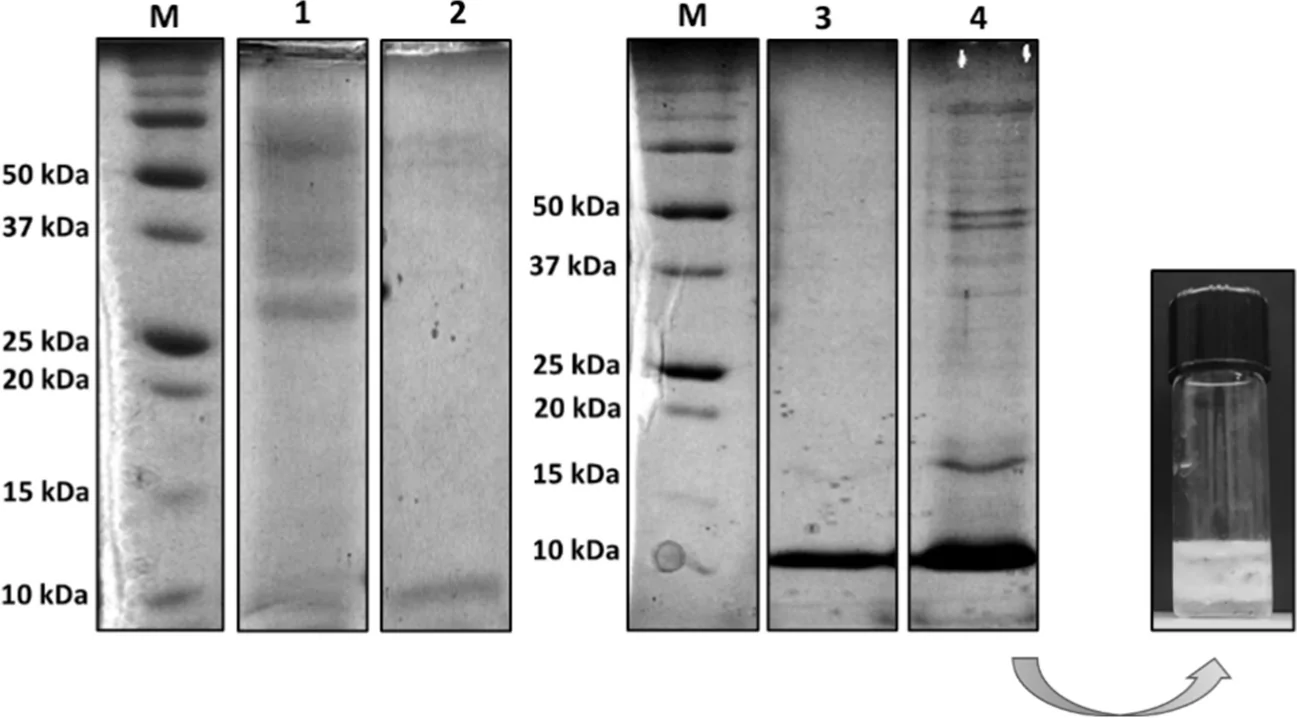
SDS-PAGE of: (1) P1 fraction from gel filtration, (2) the same fraction after treatment with urea, DTT, and IAM, (3) P2 fraction from gel filtration, (4) the same fraction after concentration, M) marker. On the right, the emulsion of P2 fraction after concentration (0.05 mg/ml)

To validate this hypothesis P2 sample (from gel filtration) was concentrated, while P1 (from gel filtration) was subjected to a harsher denaturation treatment (with urea, DTT, and IAM), and then both samples were loaded on SDS PAGE. In Fig. 8a unique band near 10 kDa, relative to P1 after urea denaturation and cys carboxyamidomethylation was detected, but its emulsification activity was lost. On the other hand, the presence of several bands at higher molecular weight raised after P2 sample concentration, and the formation of a stable emulsion further confirms that hypothesis.

## Discussion

*F. solani* is one of the few fungal species that have been proposed and studied for its plastic-degrading ability (Spina et al. 2021; Ekanayaka et al. 2022). Two strains were isolated from different landfills and herein analyzed for their BS production because it is known that secondary metabolites, such as enzymes and BS are involved in plastic degradation. We analysed both strains but very few differences between them were observed. After a rough purification procedure, we were able to separate by gel filtration a high molecular weight peak endowed with emulsification activity and a low molecular weight peak whose components were able to reduce the surface tension. These results are in agreement with the recent and generally accepted BS classification, which predicts that low molecular weight compounds are able to reduce surface tension, while high molecular weight compounds are more efficient in stabilizing emulsions. As the low molecular weight peak analysis is concerned, the preliminary analysis confirmed the presence of a cationic surfactant but did not allow its identification. Deeper studies about the nature of this surfactant will be carried on, using other techniques such as NMR spectroscopy. We then focused our attention on the high molecular weight fraction. Indeed, the unique protein identified in the raw extract, an uncharacterized protein containing the CFEM domain (common in fungal extracellular membrane), was recovered in a fraction with no emulsifying activity, while several high molecular weight proteins were observed in the emulsion active fraction. Further experiments demonstrated that these proteins arose from aggregation of the CFEM-containing protein.

CFEM, an eight-cysteine-containing domain, is predominantly comprised of hydrophobic residues (32–45% by frequency) and it was demonstrated that it is unique to fungi (Kulkarni et al. 2003). Some fungal proteins that contain this domain were proposed to play an important role in pathogenesis. For example, CFEM proteins bind heme and function in heme acquisition in the pathogenic fungus *Candida albicans*. In *Saccharomyces cerevisiae* the single CFEM protein, Ccw14, is involved in cell wall stability, and in *Aspergillus fumigatus*, deletion of all CFEM proteins also affects cell wall stability (Kornitzer and Roy 2020). These proteins do not appear to be involved in biofilm formation or hemin uptake but do play a role in cell wall integrity, possibly by stabilizing the formation of polysaccharide fibers (Vaknin et al. 2014). Therefore, the role of these proteins is still a matter of debate. It is worth noting that their length is highly variable and the CFEM domain can also occur several times in the same protein. In our case, C7Z2B1 is a small protein (104aa) with a unique CFEM domain and two additional Cys residues compared to the conserved eight of the CFEM domain. The more interesting discovery of our work was that the monomeric protein was not able to stabilize the emulsion, on the contrary, its very stable aggregates are.

**Fig. 9:**
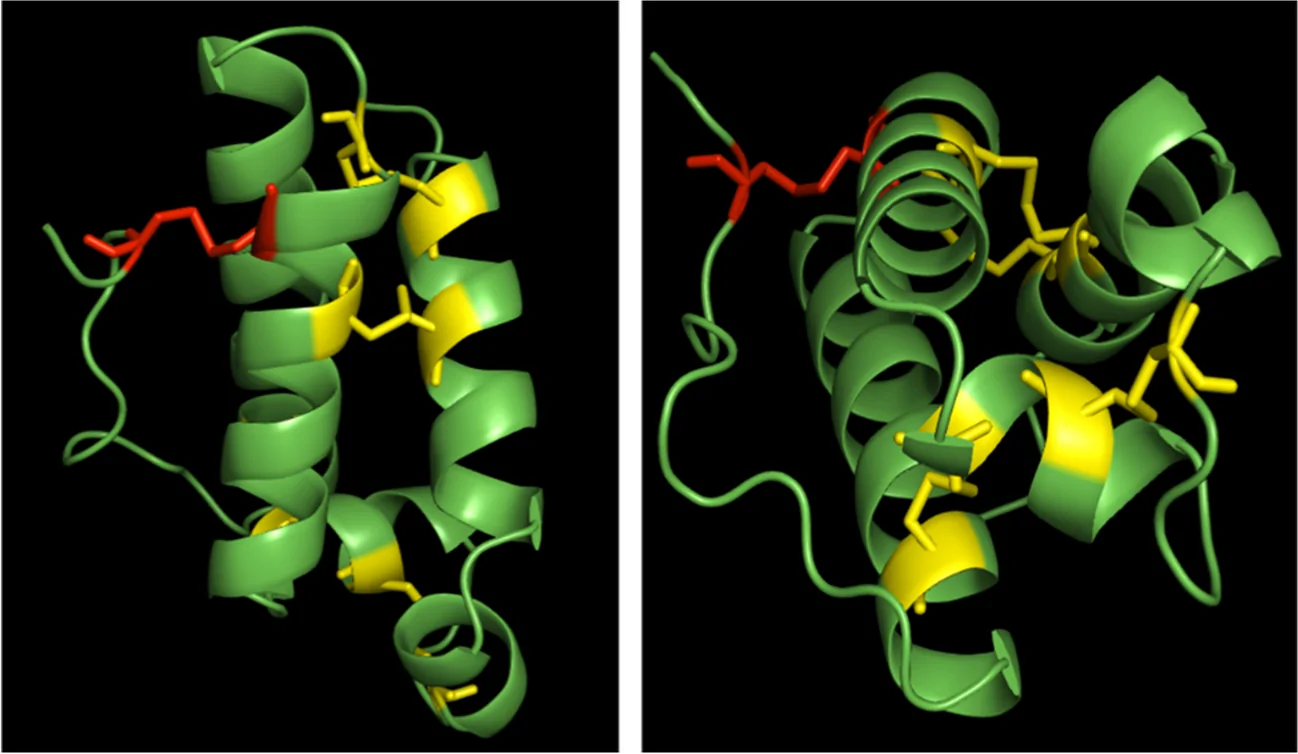
Two images of 3D protein structure predicted for C7Z2B1 by Alphafold. In yellow conserved disulfide bridges in the CFEM domain; in red the additional disulfide bridge linking the C-terminus to the α helix

An increase in the amount of the aggregate form should occur when protein concentration increases. The tendency of CFEM-containing proteins to form oligomers was already demonstrated in the case of Csa2 from *C. albicans* whose crystal structure showed the protein in a trimeric form, while the molecular weight of the protein in solution is consistent with a dimeric assembly (Nasser et al. 2016). The 3D protein structure predicted for C7Z2B1 with a high level of confidence by Alphafold (https://alphafold.ebi.ac.uk/), shows that the two additional Cys residues could form a further disulfide bridge between the C term and one of the three long alpha helixes (Fig. 9).

The observation that aggregated forms of proteins are more active in emulsion stabilization than their monomeric counterparts is not new. Milk whey proteins, for example, are largely known and recognized for their emulsification ability, which arises from aggregation formation upon heating treatment. Heating can lead to the partial unfolding of proteins and thus exposure of groups previously buried within their structure. This may ultimately result in the aggregation between different molecules through hydrophobic interactions, hydrogen bonding, and, if present, disulfide bonding, which renders the aggregation often irreversible (Amagliani and Schmitt 2017). Also, plant-derived proteins (e.g. from soy, pea, potato) show the same behavior of milky protein and their studies are gaining more interest, due to the widespread need for alternative protein sources for sustainability and food security reasons.

In conclusion, a novel functionality of the CFEM domain-containing proteins has been suggested, analyzing the activity and the predicted structure of the C7Z2B1 protein secreted by *F. solani.* These results give evidence that there might be other new classes of fungal proteins, behind the already known HPBs families, with good BS/BE properties. In this regard, the protein herein identified revealed better emulsification ability (E_24_ 80%, 0.06 mg/ml) than other known BS/BE proteins analyzed in the same condition. Indeed, the E_24_ values of other fungal proteins as SapPC, AtCp and ThCp were 70%, 83% and 70%, respectively, at 0.1 mg/ml (Cicatiello et al. 2019; Pitocchi et al. 2020). Moreover, the hydrophobin HFBII shows an E_24_ of 74% at a much higher concentration, 1 mg/ml (Blesic et al. 2018). On the other hand, the common anionic surfactant SDS showed no ability to stabilize emulsions, at a molar concentration comparable to that of the CFEM domain-containing protein (about 3 mM) (Pitocchi et al. 2023).

## Supplementary Information

Below is the link to the electronic supplementary material.

Supplementary file1 (PDF 643 KB)

## Data Availability

All data generated or analyzed during this study are included in this article and its supplementary information files.
