# Supplementary material for: The essential role of aggregation for the emulsifying ability of a fungal CYS-rich protein
Source: Appl Microbiol Biotechnol. 2024 Jun 3;108:358. doi: 10.1007/s00253-024-13182-7 (PMC11147851; doi:10.1007/s00253-024-13182-7)
Supplement: Supplementary file 1 [file 253_2024_13182_MOESM1_ESM.pdf]

Applied Microbiology and Biotechnology

Supplementary Information

THE ESSENTIAL ROLE OF AGGREGATION FOR THE EMULSIFYING ABILITY OF A FUNGAL CYS-RICH PROTEIN

Rossana Pitocchi[1], Paola Cicatiello[1]*, Anna Illiano[1], Carolina Fontanarosa[1], Federica Spina[2], Giovanna Cristina Varese[2], Angela Amoresano[1], Alessandra Piscitelli[1], Paola Giardina[1]

[1]Department of Chemical Sciences, University of Naples Federico II, Via Cintia, 80126 Naples, Italy.
[2]Department of Life Sciences and Systems Biology, University of Turin, Viale P.A. Mattioli 25, 10125 Turin, Italy
*****Correspondence: paola.cicatiello@unina.it; Tel. +3908174327

Table S1: List of precursor ion, product ions, collision energy and declustering potential parameters for the analysis of the phenolic compounds using LC-MRM-MS/MS in negative (ESI-) ionization modes. (Declastering DP and Collision Energy CE).

Precursor	Product	Dwell	Analyte	DP	CE
285.000	151.000	50.0	Kaempferol	-86.000	-28.000
285.000	92.800	50.0	Kaempferol	-86.000	-28.000
285.000	133.000	50.0	Kaempferol	-60.000	-28.000
242.800	158.900	50.0	Piceatannol	-36.000	-38.000
242.800	201.000	50.0	Piceatannol	-36.000	-38.000
431.000	268.700	50.0	Genistin	-86.000	-18.000
431.000	132.800	50.0	Genistin	-86.000	-18.000
609.000	301.000	50.0	Rutin	-60.000	-30.000
609.000	299.800	50.0	Rutin	-61.000	-50.000
609.000	270.900	50.0	Rutin	-61.000	-50.000
579.100	270.800	50.0	Naringin	-96.000	-56.000
579.100	150.800	50.0	Naringin	-96.000	-56.000
227.000	185.000	50.0	Resveratrol	-96.000	-32.000
227.000	143.000	50.0	Resveratrol	-96.000	-32.000
316.800	150.800	50.0	Myricetin	-56.000	-36.000
316.800	178.900	50.0	Myricetin	-56.000	-36.000
286.800	150.700	50.0	Eriodictyol	-31.000	-22.000
286.800	134.900	50.0	Eriodictyol	-31.000	-22.000
356.900	82.900	50.0	Matairesinol	-31.000	-36.000
356.900	137.000	50.0	Matairesinol	-31.000	-36.000
301.000	252.900	50.0	Enterodiol	-56.000	-26.000
301.000	106.000	50.0	Enterodiol	-56.000	-26.000
300.900	150.900	50.0	Quercetin	-56.000	-32.000
300.900	178.600	50.0	Quercetin	-56.000	-32.000
284.900	174.900	50.0	Cyanidin	-61.000	-32.000
284.900	240.800	50.0	Cyanidin	-61.000	-32.000
285.000	132.900	50.0	Luteolin	-61.000	-46.000
285.000	150.900	50.0	Luteolin	-61.000	-46.000
285.000	217.000	50.0	Luteolin	-60.000	-46.000
285.000	199.000	50.0	Luteolin	-60.000	-46.000
296.900	106.900	50.0	Enterolactone	-46.000	-46.000
296.900	253.000	50.0	Enterolactone	-46.000	-46.000
288.800	245.000	50.0	Catechin	-71.000	-22.000
288.800	108.900	50.0	Catechin	-71.000	-22.000
166.600	123.100	50.0	Vanillic acid	-61.000	-18.000
166.600	107.800	50.0	Vanillic acid	-61.000	-18.000
178.700	107.200	50.0	Caffeic acid	-21.000	-22.000
288.800	244.800	50.0	Epicatechin	-76.000	-22.000
288.800	109.100	50.0	Epicatechin	-76.000	-22.000
300.900	256.700	50.0	Delphinidin	-51.000	-20.000
300.900	190.800	50.0	Delphinidin	-51.000	-20.000
268.800	240.900	50.0	Pelargonidin	-61.000	-26.000

268.800	200.700	50.0	Pelargonidin	-61.000	-26.000
163.000	162.000	50.0	TranspCoumaric acid	-35.000	-20.000
163.000	119.000	50.0	TranspCoumaric acid	-35.000	-20.000
300.800	163.900	50.0	Haesperetin	-51.000	-34.000
300.800	150.900	50.0	Haesperetin	-51.000	-34.000
268.600	132.900	50.0	Genistein	-101.000	-44.000
268.600	159.000	50.0	Genistein	-101.000	-44.000
267.000	210.900	50.0	Coumestrol	-81.000	-38.000
267.000	238.800	50.0	Coumestrol	-80.000	-44.000
268.800	116.900	50.0	Apigenin	-46.000	-44.000
268.800	150.800	50.0	Apigenin	-45.000	-30.000
266.800	251.700	50.0	Formonetin	-91.000	-30.000
266.800	222.800	50.0	Formonetin	-90.000	-28.000
282.800	267.500	50.0	Glycitein	-101.000	-28.000
282.800	240.000	50.0	Glycitein	-100.000	-32.000
282.900	267.800	50.0	Biochanin A	-76.000	-28.000
282.900	238.800	50.0	Biochanin A	-76.000	-28.000
254.900	239.900	50.0	Pterosilbene	-31.000	-28.000
449.000	431.000	50.0	Luteolin6cglucoside (ISOORIENTIN)	-60.000	-30.000
449.000	285.000	50.0	Luteolin6cglucoside (ISOORIENTIN)	-60.000	-30.000
449.000	377.000	50.0	Luteolin6cglucoside (ISOORIENTIN)	-60.000	-30.000
431.000	311.000	50.0	Apigenin8cglucoside (VITEXIN)	-55.000	-30.000
431.000	269.000	50.0	Apigenin8cglucoside (VITEXIN)	-55.000	-30.000
431.000	341.000	50.0	Apigenin8cglucoside (VITEXIN)	-55.000	-30.000
464.000	301.000	50.0	Quercetin3*O*galactoside	-60.000	-38.000
464.000	463.000	50.0	Quercetin3*O*galactoside	-60.000	-38.000
594.000	593.000	50.0	Kaempferol3*O*rutinoside	-65.000	-45.000
594.000	285.000	50.0	Kaempferol3*O*rutinoside	-65.000	-45.000
316.000	300.000	50.0	Isorhamnetin	-60.000	-30.000
316.000	151.000	50.0	Isorhamnetin	-60.000	-30.000
316.000	315.000	50.0	Isorhamnetin	-60.000	-30.000
538.000	537.000	50.0	Amentoflavone	-60.000	-40.000
538.000	375.000	50.0	Amentoflavone	-60.000	-40.000
353.000	191.000	50.0	Caffeoylquinic acid derivative	-60.000	-30.000
353.000	179.000	50.0	Caffeoylquinic acid derivative	-60.000	-30.000
609.000	463.000	50.0	Quercetin rutinoside	-60.000	-30.000
609.000	300.000	50.0	Quercetin rutinoside	-60.000	-30.000
505.000	463.000	50.0	Quercetin acetylhexoside	-60.000	-30.000
505.000	445.000	50.0	Quercetin acetylhexoside	-60.000	-30.000
183.000	169.000	50.0	Methyl gallate	-60.000	-20.000
183.000	125.000	50.0	Methyl gallate	-60.000	-20.000
593.000	425.000	50.0	EGCepicatechin dimer	-60.000	-30.000
197.000	169.000	50.0	Ethyl gallate	-60.000	-20.000
197.000	125.000	50.0	Ethyl gallate	-60.000	-20.000
729.000	577.000	50.0	Procyanidin dimer gallate	-60.000	-30.000
493.000	317.000	50.0	Myricetin3*O*glucuronide	-40.000	-25.000

479.000	317.000	50.0	Myricetin3*O*glucoside	-40.000	-25.000
441.000	289.000	50.0	EC3*O*gallate	-60.000	-30.000
441.000	169.000	50.0	EC3*O*gallate	-60.000	-30.000
389.000	227.000	50.0	Cisresveratrol3*O*glucoside	-40.000	-25.000
561.000	289.000	50.0	EfisetinidolEC isomer 2	-71.000	-22.000
561.000	273.000	50.0	EfisetinidolEC isomer 3	-71.000	-22.000
745.000	457.000	50.0	EGCEGC gallate	-60.000	-20.000
745.000	169.000	50.0	EGCEGC gallate	-60.000	-20.000
619.000	457.000	50.0	EGC gallate glucoside	-60.000	-20.000
619.000	305.000	50.0	EGC gallate glucoside	-60.000	-20.000
609.000	457.000	50.0	EGC digallate	-60.000	-20.000
609.000	305.000	50.0	EGC digallate	-60.000	-30.000
455.000	289.000	50.0	EC methyl gallate	-60.000	-30.000
455.000	183.000	50.0	EC methyl gallate	-60.000	-30.000
425.000	169.000	50.0	EAfzelechin gallate	-60.000	-30.000
715.000	563.000	50.0	Theaflavin gallate	-60.000	-30.000
715.000	545.000	50.0	Theaflavin gallate	-60.000	-30.000
867.000	715.000	50.0	Theaflavin diglallate	-60.000	-30.000
867.000	563.000	50.0	Theaflavin diglallate	-60.000	-30.000
563.000	545.000	50.0	Theaflavin	-60.000	-30.000
153.100	133.000	50.0	Protocatechuic Acid	-40.000	-20.000
153.100	109.000	50.0	Protocatechuic Acid	-40.000	-20.000
153.100	93.000	50.0	Protocatechuic Acid	-40.000	-20.000
353.300	191.000	50.0	Chlorogenic Acid	-50.000	-20.000
353.300	163.000	50.0	Chlorogenic Acid	-50.000	-20.000
353.300	145.000	50.0	Chlorogenic Acid	-50.000	-20.000
353.300	179.000	50.0	Chlorogenic Acid	-50.000	-30.000
353.300	135.000	50.0	Chlorogenic Acid	-50.000	-30.000
335.300	179.000	50.0	Caffeoyliquinic Acid Lactone	-60.000	-30.000
335.300	161.000	50.0	Caffeoyliquinic Acid Lactone	-60.000	-30.000
193.200	149.000	50.0	Ferulic Acid	-40.000	-20.000
193.200	134.000	50.0	Ferulic Acid	-40.000	-20.000
591.200	283.000	50.0	Linarin	-40.000	-18.000
591.200	447.000	50.0	Linarin	-40.000	-18.000
591.200	420.000	50.0	Linarin	-40.000	-18.000
415.400	295.000	50.0	Puerarin	-40.000	-30.000
415.400	325.000	50.0	Puerarin	-40.000	-30.000
415.400	399.000	50.0	Puerarin	-40.000	-45.000
415.400	381.000	50.0	Puerarin	-40.000	-45.000
299.300	284.000	50.0	Diosmetin	-40.000	-26.000
299.300	153.000	50.0	Diosmetin	-40.000	-26.000
299.300	201.000	50.0	Diosmetin	-40.000	-26.000
299.300	55.000	50.0	Diosmetin	-40.000	-26.000
769.700	768.000	50.0	Typhanoside	-40.000	-35.000
769.700	314.000	50.0	Typhanoside	-40.000	-35.000
447.400	327.000	50.0	Astragalin	-40.000	-35.000

447.400	284.000	50.0	Astragalin	-40.000	-35.000
163.200	147.000	50.0	Coumaric Acid	-40.000	-20.000
163.200	119.000	50.0	Coumaric Acid	-40.000	-20.000
163.200	91.000	50.0	Coumaric Acid	-40.000	-20.000
337.300	191.000	50.0	3PCoumaroylquinic Acid	-40.000	-30.000
337.300	163.000	50.0	3PCoumaroylquinic Acid	-40.000	-30.000
447.300	431.000	50.0	Orientin	-40.000	-30.000
447.300	413.000	50.0	Orientin	-40.000	-30.000
447.300	357.000	50.0	Orientin	-40.000	-30.000
447.300	285.000	50.0	Orientin	-40.000	-30.000
447.400	285.000	50.0	Luteolin7*O*Glucoside	-60.000	-30.000
447.400	153.000	50.0	Luteolin7*O*Glucoside	-60.000	-30.000
447.400	60.000	50.0	Luteolin7*O*Glucoside	-60.000	-30.000
577.500	270.000	50.0	Isorhoifolin	-60.000	-40.000
577.500	269.000	50.0	Isorhoifolin	-60.000	-40.000
577.500	45.000	50.0	Isorhoifolin	-60.000	-40.000
463.400	43.000	50.0	Hyperoside	-60.000	-38.000
463.400	301.000	50.0	Hyperoside	-60.000	-38.000
447.400	301.000	50.0	Quercetin*O*Rhamnoside	-60.000	-30.000
447.400	431.000	50.0	Quercetin*O*Rhamnoside	-60.000	-30.000
461.900	299.000	50.0	Quercetin*O*Hexoside	-60.000	-30.000
593.500	285.000	50.0	Nicotinflorin	-60.000	-45.000
593.500	257.000	50.0	Nicotinflorin	-60.000	-45.000
593.500	449.000	50.0	Nicotinflorin	-60.000	-45.000
431.100	285.000	50.0	Kaempeferol3*O*Rhamnoside	-60.000	-30.000
271.200	153.000	50.0	Naringenin	-60.000	-30.000
271.200	177.000	50.0	Naringenin	-60.000	-30.000
271.200	119.000	50.0	Naringenin	-60.000	-30.000
271.200	107.000	50.0	Naringenin	-60.000	-30.000
271.200	93.000	50.0	Naringenin	-60.000	-30.000
271.200	83.000	50.0	Naringenin	-60.000	-30.000
433.400	271.000	50.0	Naringenin7*O*Glucoside	-60.000	-20.000
433.400	295.000	50.0	Naringenin7*O*Glucoside	-60.000	-20.000
433.400	270.000	50.0	Naringenin7*O*Glucoside	-60.000	-20.000
579.500	271.000	50.0	Naringenin7*O*Neohesperidoside	-80.000	-35.000
579.500	459.000	50.0	Naringenin7*O*Neohesperidoside	-80.000	-35.000
623.500	314.000	50.0	Isorhamentin3*O*Neohesperidoside	-80.000	-43.000
179.200	67.000	50.0	Theobromine	-45.000	-30.000
179.200	109.000	50.0	Theobromine	-45.000	-30.000
179.200	83.000	50.0	Theobromine	-45.000	-30.000
179.200	69.000	50.0	Theobromine	-45.000	-30.000
179.200	138.000	50.0	Theobromine	-45.000	-30.000
193.200	109.000	50.0	Caffeine	-45.000	-30.000
193.200	123.000	50.0	Caffeine	-45.000	-30.000
193.200	153.000	50.0	Caffeine	-45.000	-30.000
193.200	60.000	50.0	Procynadin B2	-45.000	-22.000

135.200	54.000	50.0	Tetramethyl Pyrazine	-45.000	-22.000
135.200	80.000	50.0	Tetramethyl Pyrazine	-45.000	-22.000
135.200	121.000	50.0	Tetramethyl Pyrazine	-45.000	-22.000
135.200	96.000	50.0	Tetramethyl Pyrazine	-45.000	-22.000
121.200	54.000	50.0	Trimethyl Pyrazine	-45.000	-22.000
121.200	42.000	50.0	Trimethyl Pyrazine	-45.000	-22.000
121.200	39.000	50.0	Trimethyl Pyrazine	-45.000	-22.000
865.800	289.000	50.0	Procynadin C1	-45.000	-22.000
865.800	393.000	50.0	Procynadin C1	-45.000	-22.000
865.800	713.000	50.0	Procynadin C1	-45.000	-22.000
865.800	849.000	50.0	Procynadin C1	-45.000	-22.000
865.800	577.000	50.0	Procynadin C1	-45.000	-22.000
577.500	427.000	50.0	Procynadin B2	-45.000	-22.000
577.500	291.000	50.0	Procynadin B2	-45.000	-22.000
577.500	561.000	50.0	Procynadin B2	-45.000	-22.000
575.500	109.000	50.0	Proanthocyanidin A2	-45.000	-22.000
575.500	139.000	50.0	Proanthocyanidin A2	-45.000	-22.000
575.500	559.000	50.0	Proanthocyanidin A2	-45.000	-22.000
169.000	52.000	50.0	Gallic acid	-64.000	-45.000
169.000	125.000	50.0	Gallic acid	-64.000	-20.000
169.000	80.000	50.0	Gallic acid	-64.000	-30.000
169.000	126.000	50.0	Gallic acid	-49.000	-21.000
169.000	124.900	50.0	Gallic acid	-66.000	-20.000
169.000	106.800	50.0	Gallic acid	-66.000	-18.000

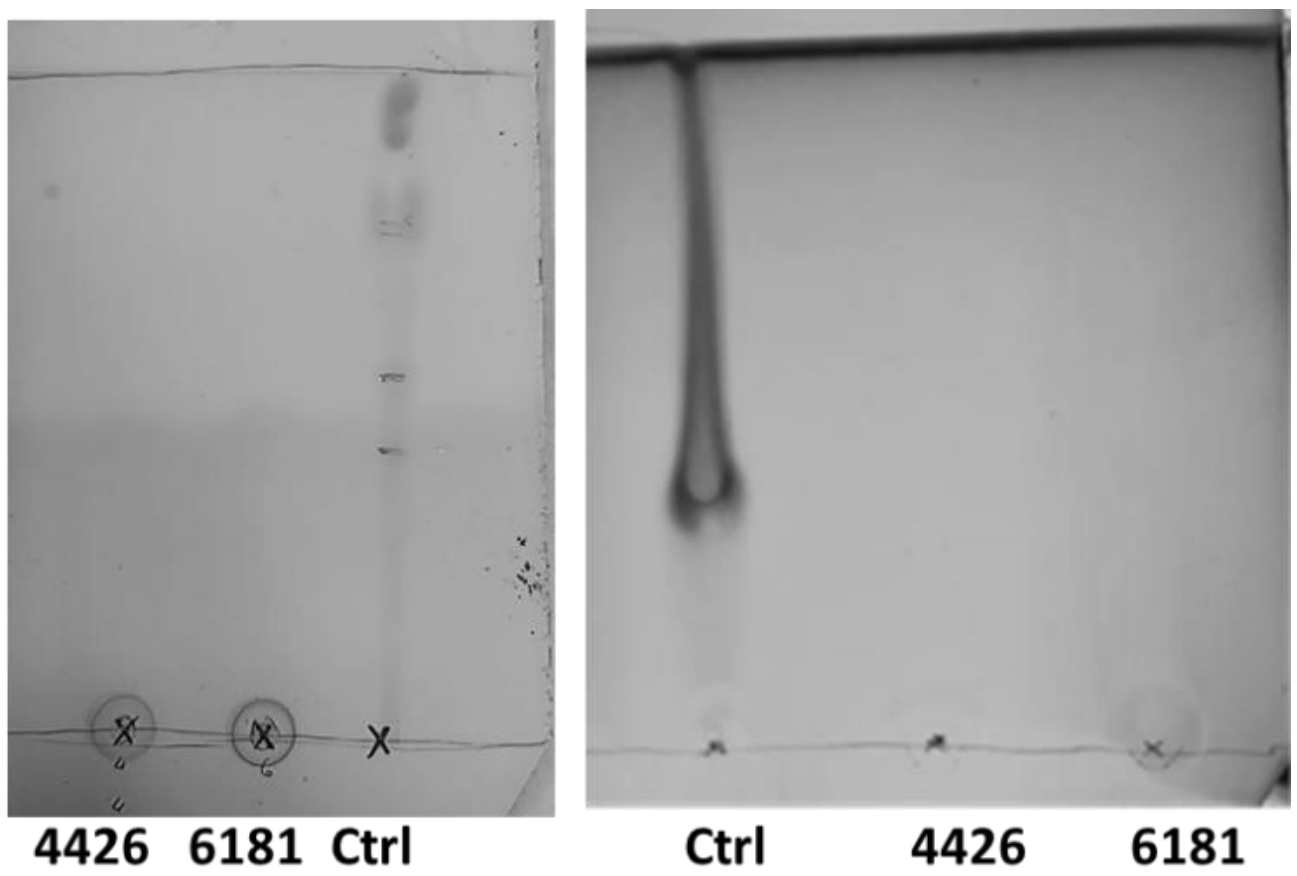

Fig S1: TLC of the extracted broths, stained by phosphomolybdic acid (10% w/v EtOH) for lipids detection on the right, and sulfuric acid (3% v/v) for sugars detection on the left. Olive oil was used as a control in both experiments.

A)

FS 4426		
**Identified peptides**	**Protein sequence coverage**	**Protein accession number**
K.TCTEANMFLCMCKIK.A	41%	C7Z2B1_NECH7<br>gi|302897631<br>**C7Z2B1**
K.IKALTLAYR.D		
K.DCACSSCLTPQSKLDAIATGK.D		
K.ALTLAYR.D		
K.ALTLAYRDCACSSCLTPQSK.L		
K.LDAIATGK.D		

FS 6181		
**Identified peptides**	**Protein sequence coverage**	**Protein accession number**
K. DICNQYQAPVAWLPDTCPA	47%	C7Z2B1_NECH7<br>gi|302897631<br>**C7Z2B1**
K. LDAIATGK.D		
K.DCACSSCLTPQSK.L		
K.ALTLAYR.D		

B)

MKVSAFLSSVAVTLASIGSANAATPLCAITCFTAVMNHEAAKTCTEANMFLCM
CKIKALTLAYRDCACSSCLTPQSKLDAIATGKDICNQYQAPVAWLPDTCPA

C)

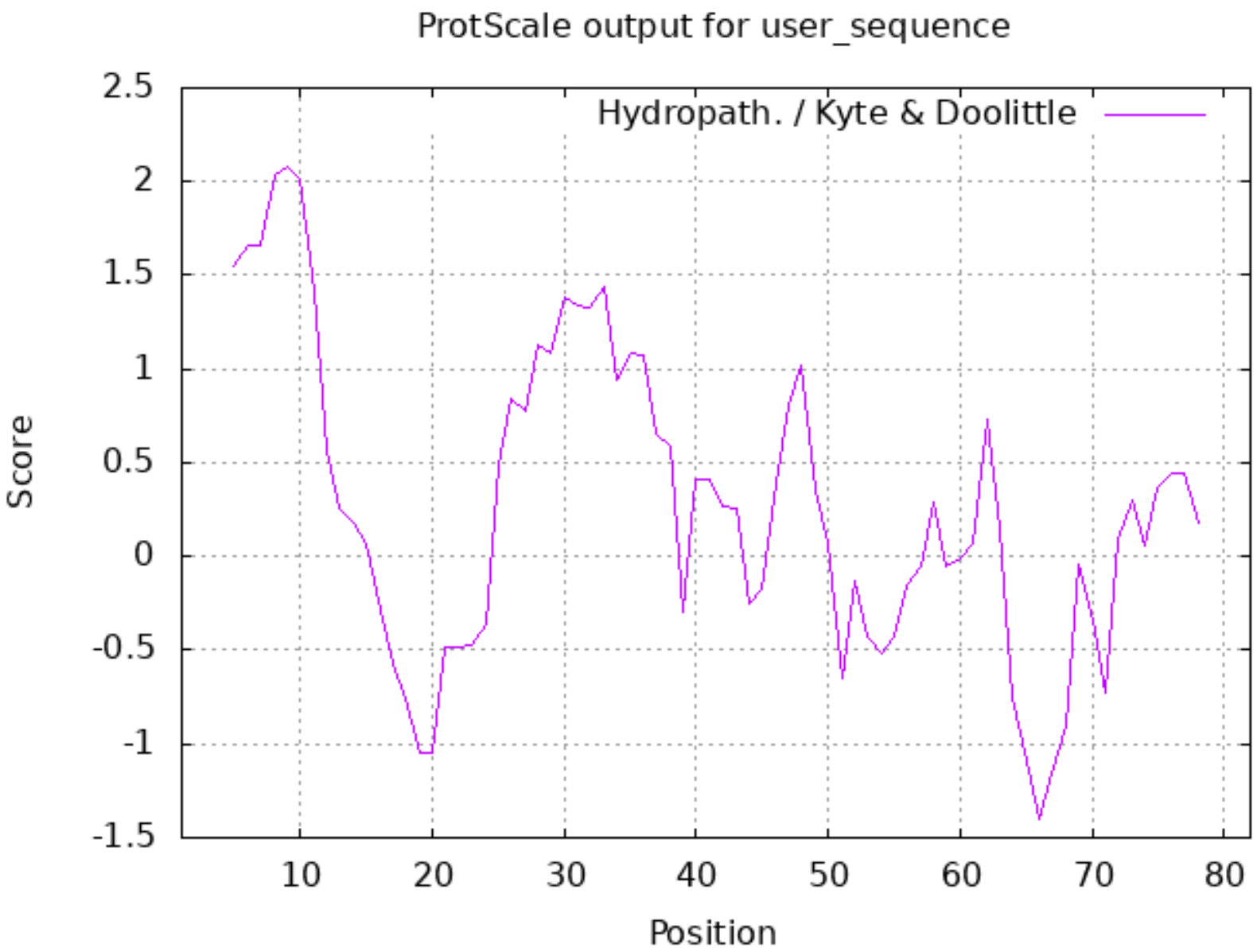

Fig. S2: A) Overview tables of protein identification. B) Identified protein sequence. ; the Cys typical of CFEM domain are highlighted in yellow, the additional Cys in red. C) Hydropathy plot of the identified protein (without signal peptide) obtained with Expasy protScale tool.

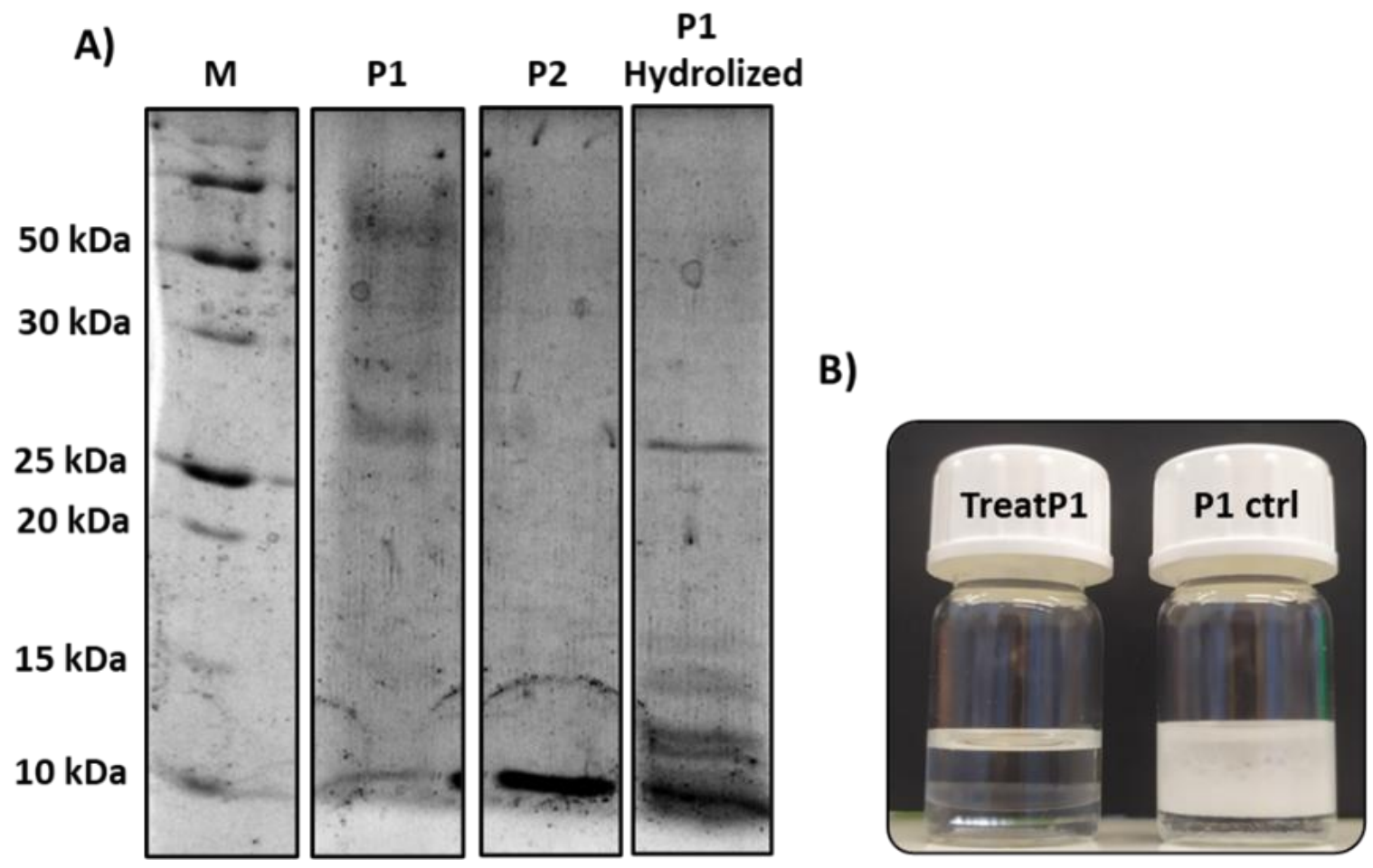

Fig. S3: A) SDS-PAGE of P1 after incubation with proteinase K (P1 hydrolized); P1 and P2 were loaded as controls. B) emulsion formed after (Treat P1) and before (P1 ctrl) hydrolysis with Proteinase K.

Table S2: List of the polyphenols detected in the chromatographic peaks successive gel chromatography, with LC-MS/MS, MRM mode. The standard used to perform the quantitative analysis is gallic acid. (nd: not detected)

	**P1**	**P2**	**P3**
**Poliphenol**	**µg/L**		
**Kaempferol**	12.37	8.34	nd
**Rutin**	8.45	5.59	13.37
**Quercetin**	7.95	2.21	78.37
**Cyanidin**	8.84	0.36	177.9
**Luteolin**	41.64	2.91	23.75
**Delphinidin**	nd	nd	363.64
**Pelargonidin**	nd	nd	nd
**Haesperetin**	5.52	1.62	46.65
**luteolin$_6$cglucoside (ISOORIENTIN)**	8.29	6.63	150.42
**Quercetin acetylhexoside**	2.64	2.09	15.35
**EGCEGC gallate**	0.06	0.86	2.88
**EGC gallate glucoside**	0.69	0.37	5.58
**Theaflavin gallate**	1.04	0.95	17.89
**Theaflavin diglallate**	2.03	1.35	9.15
**Chlorogenic Acid**	6.77	34.31	617.9
**Linarin**	2.24	1.66	10.5
**Diosmetin**	2.03	6.09	226.72
**3P-Coumaroylquinic Acid**	nd	nd	128.45
**Orientin**	nd	1.5	27.47
**Luteolin$_7$O-Glucoside**	3.13	1.35	nd
**Nicotinflorin**	0.61	0.31	22.19
**Naringenin**	9.3	32.27	**5645.34**
**Naringenin$_7$O-Glucoside**	nd	1.29	19.75
**Procynadin C_1**	4.42	3.1	4.65
**Proanthocyanidin A_2**	0.74	0.55	3.01

Identified peptides	Sequence coverage	Unique peptides	Score sequest
LDAIATGKDICNQYQAPVAWLPDTCPA	58%	7	33.05
DCACSSCLTPQSK			
TCTEANMFLCMCK			
ALTLAYRDCACSSCLTPQSK			
LDAIATGK			
ATLAYR			
DICNQYQAPVAWLPDTCPA			

Identified peptides	Sequence coverage	Unique peptides	Score sequest
TCTEANMFLCMCK	40%	6	51.65
ALTLAYRDCACSSCLTPQSK			
TCTEANMFLCMCK			
DCACSSCLTPQSK			
DCACSSCLTPQSKLDAIATGK			
LDAIATGK			
ALTLAYR			

Fig. S4: Identification of proteins presents in P2*, after anionic exchange. The same protein initially identified, C7Z2B1, was found within two independent experiments, with a good sequence coverage and number of peptides.
